# Supplementary material for: Variation in wood density across South American tropical forests
Source: Nat Commun. 2025 Mar 10;16:2351. doi: 10.1038/s41467-025-56175-4 (PMC11893774; doi:10.1038/s41467-025-56175-4)
Supplement: Supplementary file 1 — Supplementary Information [file 41467_2025_56175_MOESM1_ESM.docx]

**Supporting information for Variation in wood density across South American tropical forests**

Martin J. P. Sullivan^1,2^, Oliver L. Phillips^2^, David Galbraith^2^, Everton Almeida^3^, Edmar Almeida de Oliveira^4^, Jarcilene Almeida^5^, Esteban Álvarez Dávila^6^, Luciana F. Alves^7^, Ana Andrade^8^, Luiz Aragão^9^, Alejandro Araujo-Murakami^10^, Eric Arets^11^, Luzmila Arroyo^12^, Omar Aurelio Melo Cruz^13^, Fabrício Baccaro^14^, Timothy R. Baker^2^, Olaf Banki^15^, Christopher Baraloto^16^, Jos Barlow^17^, Jorcely Barroso^18^, Erika Berenguer^19,17^, Lilian Blanc^20,21^, Cecilia Blundo^22^, Damien Bonal^23^, Frans Bongers^24^, Kauane Maiara Bordin^25^, Roel J. W. Brienen^2^, Igor S. Broggio^26,27^, Benoit Burban^28^, George Cabral^5^, José Luís Camargo^29^, Domingos Cardoso^30,31^, Maria Antonia Carniello^32^, Wendeson Castro^33^, Haroldo Cavalcante de Lima^30^, Larissa Cavalheiro^34,35^, Sabina Cerruto Ribeiro^36^, Sonia Cesarina Palacios Ramos^37^, Victor Chama Moscoso^38^, Jerôme Chave^39^, Fernanda Coelho^2,40^, James A. Comiskey^41,42^, Fernando Cornejo Valverde^43^, Flávia Costa^44^, Italo Antônio Cotta Coutinho^45^, Antonio Carlos Lola da Costa^46^, Marcelo Brilhante de Medeiros^47^, Jhon del Aguila Pasquel^48,49^, Géraldine Derroire^50^, Kyle Dexter^51,52,53^, Mat Disney^54^, Mário M. do Espírito Santo^55^, Tomas F. Domingues^56^, Aurélie Dourdain^50^, Alvaro Duque^57^, Cristabel Durán Rangel^58^, Fernando Elias^59,60^, Adriane Esquivel-Muelbert^61^, William Farfan-Rios^62^, Sophie Fauset^63^, Ted Feldpausch^64^, G. Wilson Fernandes^65^, Joice Ferreira^66^, Yule Roberta Ferreira Nunes^67^, João Carlos Gomes Figueiredo^55^, Karina Garcia Cabreara^68^, Roy Gonzalez^69^, Lionel Hernández^70^, Rafael Herrera^71^, Eurídice N. Honorio Coronado^72^, Walter Huaraca Huasco^19^, Mariana Iguatemy^73^, Carlos A. Joly^74^, Michelle Kalamandeen^2^, Timothy Killeen^75^, Joice Klipel^76^, Bente Klitgaard^77^, Susan G. Laurance^78,79^, William F. Laurance^78,79^, Aurora Levesley^2^, Simon L. Lewis^2,54^, Maurício Lima Dan^80^, Gabriela Lopez-Gonzalez^2^, William Magnusson^81^, Yadvinder Malhi^19^, Lucio Malizia^82^, Augustina Malizia^22^, Angelo Gilberto Manzatto^83,84^, Jose Luis Marcelo Peña^85^, Beatriz S. Marimon^86^, Ben Hur Marimon Junior^86^, Johanna Andrea Martínez-Villa^87^, Simone Matias Reis^86,36^, Thiago Metzker^88^, William Milliken^89^, Abel Monteagudo-Mendoza^90^, Peter Moonlight^91,92^, Paulo S. Morandi^4^, Pamela Moser^93^, Sandra C. Müller^76^, Marcelo Nascimento^94^, Daniel Negreiros^65^, Adriano Nogueira Lima^44^, Percy Núñez Vargas^95^, Washington L. Oliveira^93^, Walter Palacios^96^, Nadir C. Pallqui Camacho^38,2^, Alexander Parada Gutierrez^10^, Guido Pardo Molina^97^, Karla Maria Pedra de Abreu^98^, Marielos Peña-Claros^24^, Pablo José Francisco Pena Rodrigues^99^, R. Toby Pennington^92,100^, Georgia C. Pickavance^2^, John Pipoly^101,102^, Nigel C. A. Pitman^103^, Maureen Playfair^104^, Aline Pontes-Lopes^9^, Lourens Poorter^24^, Nayane Cristina Candida dos Santos Prestes^4^, Hirma Ramírez-Angulo^105^, Maxime Réjou-Méchain^106^, Carlos Reynel Rodriguez^107^, Gonzalo Rivas-Torres^108^, Priscyla M. S. Rodrigues^109^, Domingos de Jesus Rodrigues^34^, Thaiane Rodrigues de Sousa^44^, José Roberto Rodrigues Pinto^110^, Gina M. Rodriguez M.^111^, Katherine Roucoux^72^, Kalle Ruokolainen^112^, Casey M. Ryan^113^, Norma Salinas Revilla^114^, Rafael Salomão^115,116^, Rubens M. Santos^117^, Tiina Sarkinen^118^, Andressa Scabin^119^, Rodrigo Scarton Bergamin^120^, Juliana Schietti^44^, Milton Serpa de Meira Junior^110^, Julio Serrano^121^, Miles Silman^68^, Richarlly C. Silva^122^, Camila V.J. Silva^17,40,123^, Jhonathan Oliveria Silva^109^, Marcos Silveira^124^, Marcelo F. Simon^47^, Yahn Carlos Soto-Shareva^125^, Priscila Souza^126^, Rodolfo Souza^127,128^, Tereza Sposito^129^, Joey Talbot^130^, Hans ter Steege^15,131^, John Terborgh^132^, Raquel Thomas^133^, Marisol Toledo^134^, Armando Torres-Lezama^105^, William Trujillo^135^, Peter van der Hout^136^, Maria das Dores Magalhães Veloso^137^, Simone A Vieira^138^, Emilio Vilanova^139^, Jeanneth M. Villalobos Cayo^140,141^, Dora M. Villela^142^, Laura Jessica Viscarra^10^, Vincent A. Vos^143^, Verginia Wortel^144^, Francoise Yoko Ishida^79,145^, Pieter A. Zuidema^24^, Joeri A. Zwerts^146,147^

**
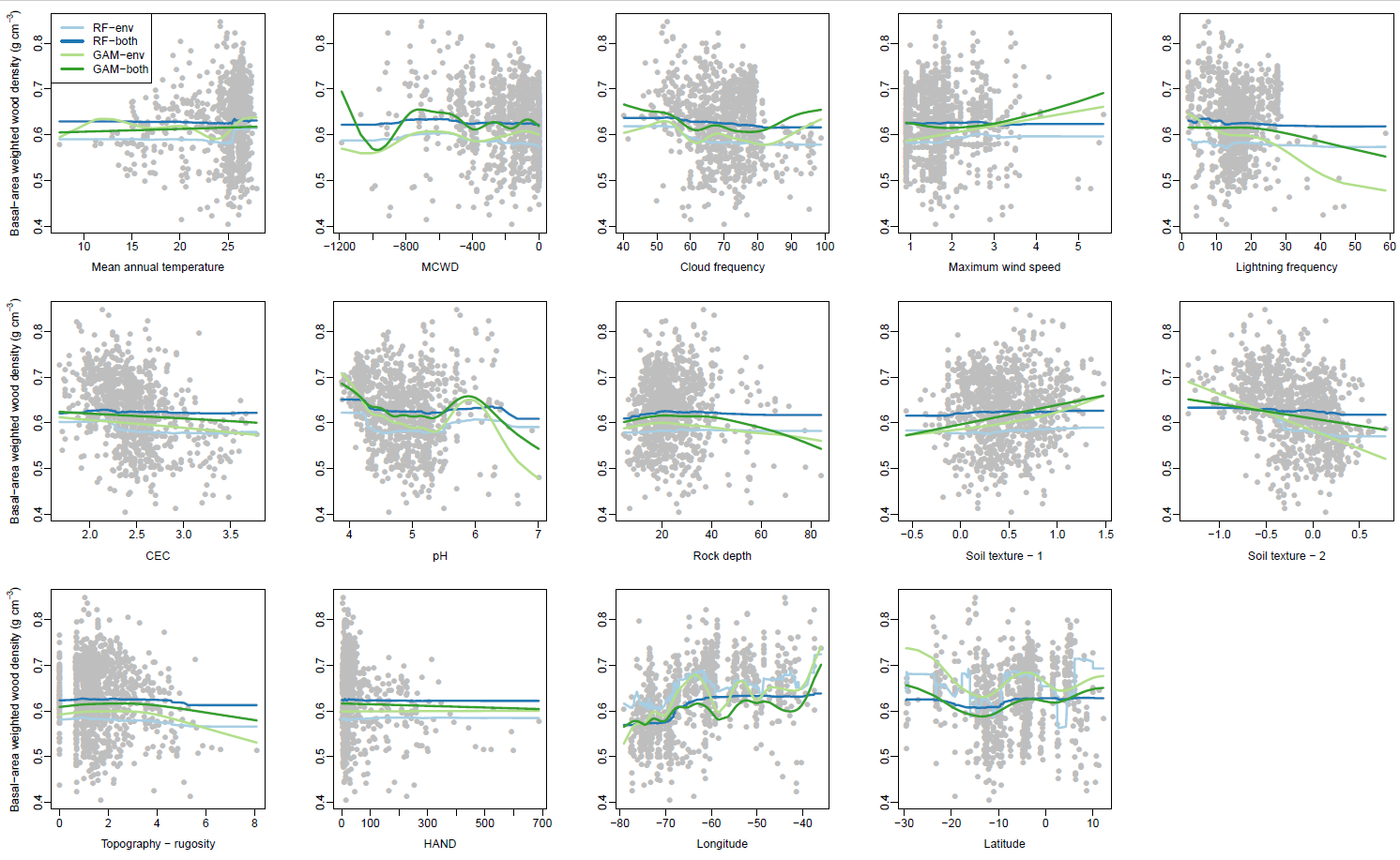
**

**Figure S1. Relationship between wood density and environmental and spatial explanatory variables.** Modelled relationships (varying the explanatory variable in question and holding other variables at their mean) are shown for GAMs (green) and random forests (blue). Paler shades indicate relationships from models with just environmental or just spatial variables, and darker shades indicate relationships from models with both sets of variables. Note that CEC has been log-transformed and rugosity square-root transformed. N = 981 plots.


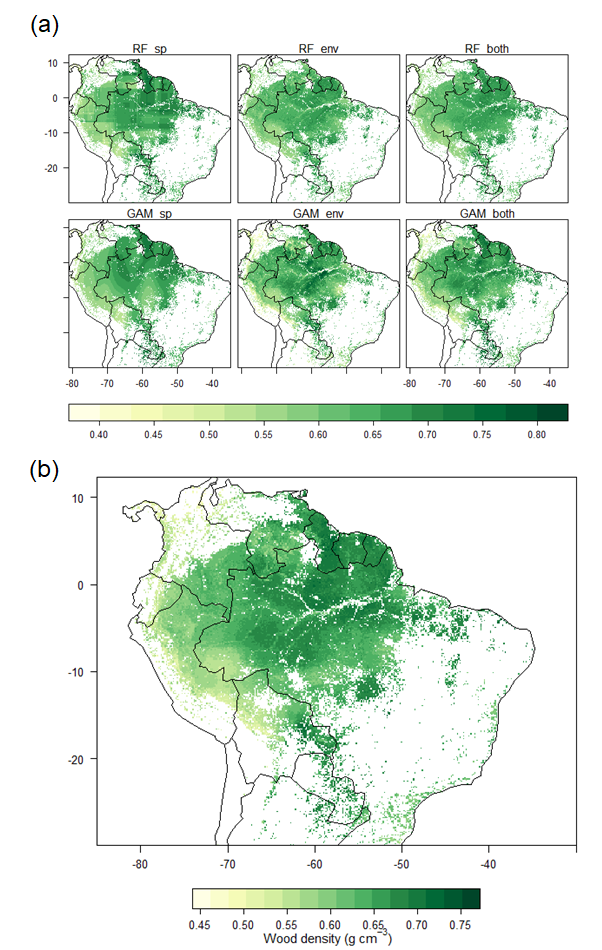


**Figure S2. Predictions of spatial variation in wood density across tropical and sub-tropical South America.** Predictions are shown from (a) random forest (RF) and generalised additive models (GAM) using spatial (sp), environmental (env) or both spatial and environmental (both) variables and (b) the averaged across the ensemble of models.


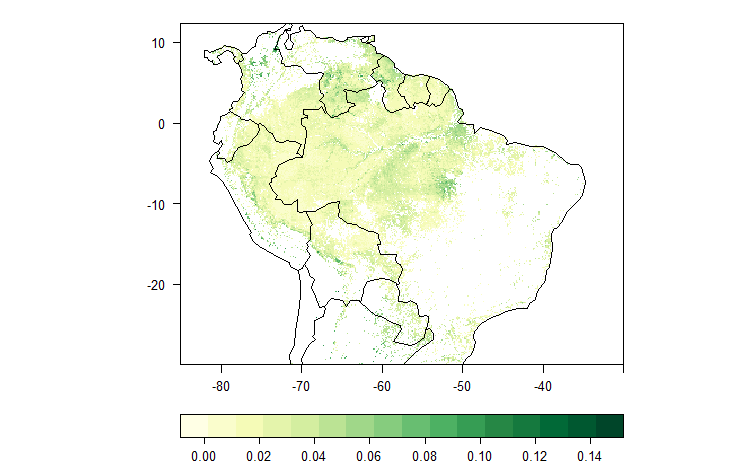


**Figure S3. Uncertainty in predictions of spatial variation in wood density.** The standard deviation of predictions between the six different models are shown, with higher values indicating greater differences in predicted wood density between models.


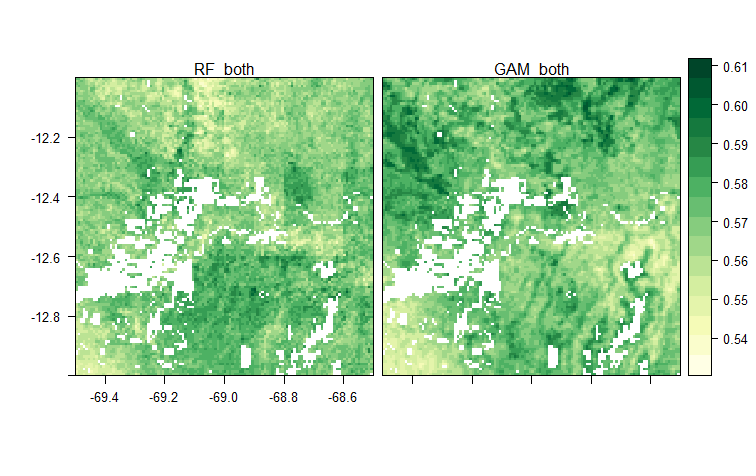
**Figure S4. Local-scale predictions of spatial variation in wood density.** Predictions have been made at 1km resolution from the random forest model (RF_both) and GAM model (GAM_both) with both spatial and environmental explanatory variables in a one degree area centred on Tambopata Natural Reserve, in Amazonian Peru.


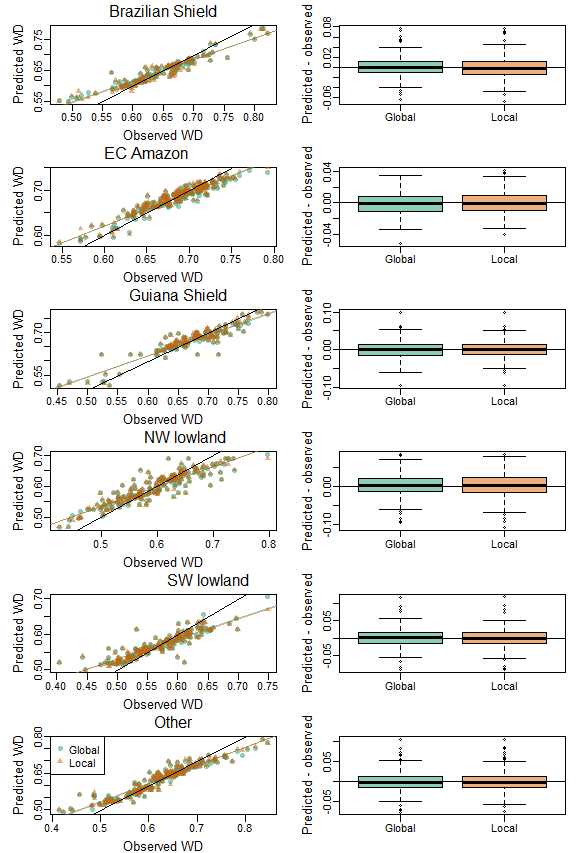


**Figure S5. Effect of fitting models within region (local) compared to across the entire dataset (global) on model predictions.** (Left) relationship between observed and predicted wood density. The black line shows the one-to-one relationship, while green and orange lines show actual relationships for global and local models respectively. (Right) difference between globally and locally fitted model predictions and observed values. N = 981 plots (Lowland-NW = 182 plots, Lowland-SW = 168, East-central Amazon = 205, Guiana Shield = 123, Brazilian Shield = 119, Other = 184). Boxplots show the median (thick line), upper and lower quartile (box) and the range of the data (whiskers, with observations >1.5 times the interquartile range away from the upper or lower quartile shown with individual points).


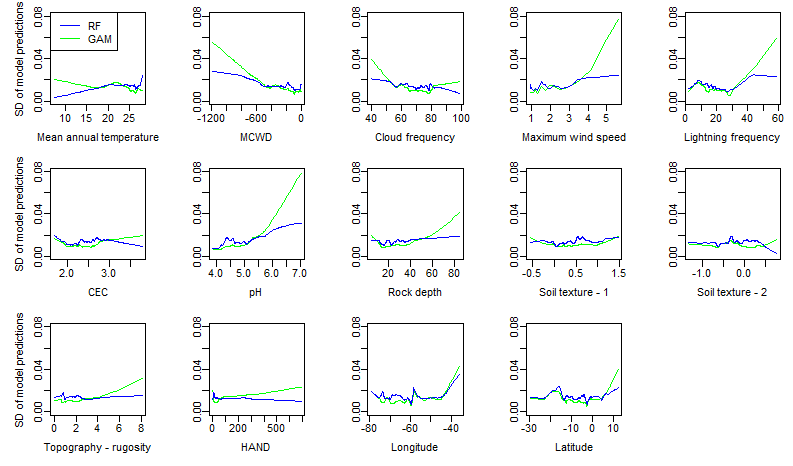


**Figure S6. Sensitivity of model predictions to subsampling the training data.** Lines show the standard deviation of model predictions (random forest, RF, and generalised additive model, GAM, both using both environmental and spatial variables) produced from 1000 random subsamples of half the training data. Lines are locally weighted polynomial smoothers relating the standard deviation of model predictors to each explanatory variable.

**Figure S7. Model prediction applicability.** (a) Location of sampling plots. (b) Multivariate dissimilarity index (DI), with values over one indicate grater dissimilarity to the most similar training data point than the average pairwise dissimilarity within the training data (96.7% of pixels have DI ≤ 1). (c-f) applicability of models based on (c) environmental variables being within the range where predictions are robust to subsampling data, (d) environmental variables being within the range observed in the training data, (e) area of applicability defined following [45] for DI values tested with spatial cross-validation and (f) for non-spatial cross-validation. For c-f, green indicates areas where models are applicable, grey indicates areas where models are not applicable, and white indicates non-forested areas.


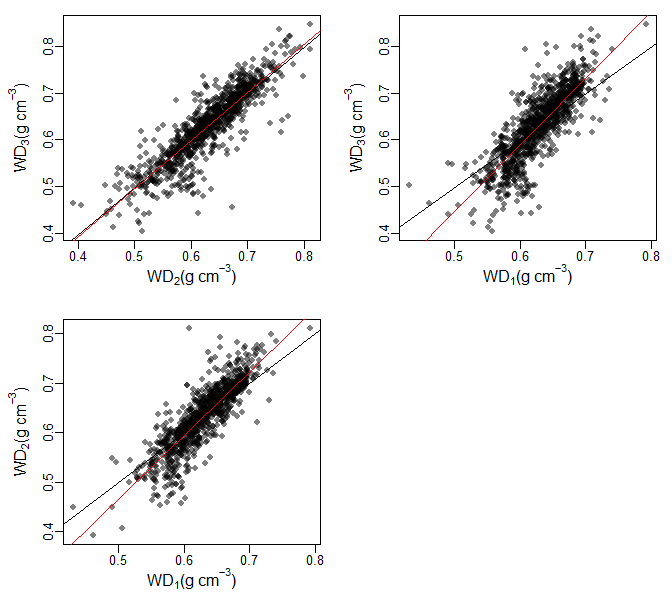


**Figure S8. Relationship between different metrics of stand-level wood density**. Wood density was calculated as the basal-area weighted mean (WD_3_), abundance weighted mean (WD_2_) or the mean of species’ wood density values discounting abundance (WD_1_). The red line shows the relationship between the two variables, and the black line shows a one-to-one relationship. Metrics are positively correlated: WD_3_-WD_2_, r=0.89, WD_3_-WD_1_, r=0.82, WD_2_-WD_1_, r=0.86).


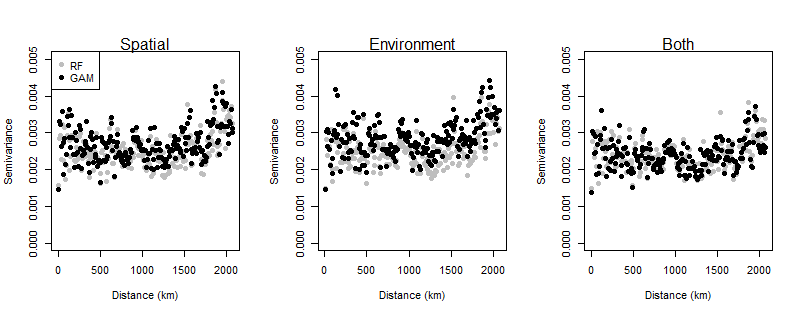


**Figure S9. Assessment of residual spatial autocorrelation.** This has been done for random forest (RF) and generalised additive models (GAM) constructed with spatial, environmental or both spatial and environmental explanatory variables.
