# Supplementary material for: Variation in wood density across South American tropical forests
Source: Nat Commun. 2025 Mar 10;16:2351. doi: 10.1038/s41467-025-56175-4 (PMC11893774; doi:10.1038/s41467-025-56175-4)
Supplement: Supplementary file 2 — Reporting Summary [file 41467_2025_56175_MOESM2_ESM.pdf]

nature portfolio

Corresponding author(s): Martin Sullivan

Last updated by author(s): Oct 2, 2024

Reporting Summary

Nature Portfolio wishes to improve the reproducibility of the work that we publish. This form provides structure for consistency and transparency in reporting. For further information on Nature Portfolio policies, see our Editorial Policies and the Editorial Policy Checklist.

Statistics

For all statistical analyses, confirm that the following items are present in the figure legend, table legend, main text, or Methods section.

n/a	Confirmed	
☐	☒	The exact sample size (*n*) for each experimental group/condition, given as a discrete number and unit of measurement
☐	☒	A statement on whether measurements were taken from distinct samples or whether the same sample was measured repeatedly
☒	☐	The statistical test(s) used AND whether they are one- or two-sided<br>*Only common tests should be described solely by name; describe more complex techniques in the Methods section.*
☐	☒	A description of all covariates tested
☐	☒	A description of any assumptions or corrections, such as tests of normality and adjustment for multiple comparisons
☐	☒	A full description of the statistical parameters including central tendency (e.g. means) or other basic estimates (e.g. regression coefficient) AND variation (e.g. standard deviation) or associated estimates of uncertainty (e.g. confidence intervals)
☒	☐	For null hypothesis testing, the test statistic (e.g. *F*, *t*, *r*) with confidence intervals, effect sizes, degrees of freedom and *P* value noted<br>*Give P values as exact values whenever suitable.*
☒	☐	For Bayesian analysis, information on the choice of priors and Markov chain Monte Carlo settings
☒	☐	For hierarchical and complex designs, identification of the appropriate level for tests and full reporting of outcomes
☒	☐	Estimates of effect sizes (e.g. Cohen's *d*, Pearson's *r*), indicating how they were calculated

Our web collection on statistics for biologists contains articles on many of the points above.

Software and code

Policy information about availability of computer code

Data collection	No software was used for data collection.
Data analysis	Data were processed and analysed in R using functions in the BIOMASS, raster, mgcv, randomForest and fastshap R packages. The R code used to run analyses and produce figures are deposited at https://doi.org/10.5521/forestplots.net/2024_4.

For manuscripts utilizing custom algorithms or software that are central to the research but not yet described in published literature, software must be made available to editors and reviewers. We strongly encourage code deposition in a community repository (e.g. GitHub). See the Nature Portfolio guidelines for submitting code & software for further information.

Data

Policy information about availability of data

All manuscripts must include a data availability statement. This statement should provide the following information, where applicable:

- Accession codes, unique identifiers, or web links for publicly available datasets
- A description of any restrictions on data availability
- For clinical datasets or third party data, please ensure that the statement adheres to our policy

Plot-level wood density values and extracted environmental variables that support this work are deposited at https://doi.org/10.5521/forestplots.net/2024_4. Model predictions and measures of uncertainty are deposited at 10.6084/m9.figshare.27118437.

Research involving human participants, their data, or biological material

Policy information about studies with human participants or human data. See also policy information about sex, gender (identity/presentation), and sexual orientation and race, ethnicity and racism.

Reporting on sex and gender	Research does not involve human participants
Reporting on race, ethnicity, or other socially relevant groupings	Research does not involve human participants
Population characteristics	Research does not involve human participants
Recruitment	Research does not involve human participants
Ethics oversight	Research does not involve human participants

Note that full information on the approval of the study protocol must also be provided in the manuscript.

Field-specific reporting

Please select the one below that is the best fit for your research. If you are not sure, read the appropriate sections before making your selection.

☐ Life sciences ☐ Behavioural & social sciences ☒ Ecological, evolutionary & environmental sciences

For a reference copy of the document with all sections, see nature.com/documents/nr-reporting-summary-flat.pdf

Life sciences study design

All studies must disclose on these points even when the disclosure is negative.

Sample size	*Describe how sample size was determined, detailing any statistical methods used to predetermine sample size OR if no sample-size calculation was performed, describe how sample sizes were chosen and provide a rationale for why these sample sizes are sufficient.*
Data exclusions	*Describe any data exclusions. If no data were excluded from the analyses, state so OR if data were excluded, describe the exclusions and the rationale behind them, indicating whether exclusion criteria were pre-established.*
Replication	*Describe the measures taken to verify the reproducibility of the experimental findings. If all attempts at replication were successful, confirm this OR if there are any findings that were not replicated or cannot be reproduced, note this and describe why.*
Randomization	*Describe how samples/organisms/participants were allocated into experimental groups. If allocation was not random, describe how covariates were controlled OR if this is not relevant to your study, explain why.*
Blinding	*Describe whether the investigators were blinded to group allocation during data collection and/or analysis. If blinding was not possible, describe why OR explain why blinding was not relevant to your study.*

Behavioural & social sciences study design

All studies must disclose on these points even when the disclosure is negative.

Study description	*Briefly describe the study type including whether data are quantitative, qualitative, or mixed-methods (e.g. qualitative cross-sectional, quantitative experimental, mixed-methods case study).*
Research sample	*State the research sample (e.g. Harvard university undergraduates, villagers in rural India) and provide relevant demographic information (e.g. age, sex) and indicate whether the sample is representative. Provide a rationale for the study sample chosen. For studies involving existing datasets, please describe the dataset and source.*
Sampling strategy	*Describe the sampling procedure (e.g. random, snowball, stratified, convenience). Describe the statistical methods that were used to predetermine sample size OR if no sample-size calculation was performed, describe how sample sizes were chosen and provide a rationale for why these sample sizes are sufficient. For qualitative data, please indicate whether data saturation was considered, and what criteria were used to decide that no further sampling was needed.*
Data collection	*Provide details about the data collection procedure, including the instruments or devices used to record the data (e.g. pen and paper, computer, eye tracker, video or audio equipment) whether anyone was present besides the participant(s) and the researcher, and whether the researcher was blind to experimental condition and/or the study hypothesis during data collection.*
Timing	*Indicate the start and stop dates of data collection. If there is a gap between collection periods, state the dates for each sample cohort.*

Data exclusions	*If no data were excluded from the analyses, state so OR if data were excluded, provide the exact number of exclusions and the rationale behind them, indicating whether exclusion criteria were pre-established.*
Non-participation	*State how many participants dropped out/declined participation and the reason(s) given OR provide response rate OR state that no participants dropped out/declined participation.*
Randomization	*If participants were not allocated into experimental groups, state so OR describe how participants were allocated to groups, and if allocation was not random, describe how covariates were controlled.*

Ecological, evolutionary & environmental sciences study design

All studies must disclose on these points even when the disclosure is negative.

Study description	The purpose of the study was to model spatial variation in wood density as a function of spatial and environmental variables, and to use these models to predict wood density across tropical and sub-tropical South American forests. Plots were established in areas of forest, and all stems of 10cm diameter at breast height or above were measured (giving their diameter, from which basal area can be calculated) and identified to species wherever possible. The taxonomic identify of each stem was used to look up their wood density using published values. The basal-area weighted mean of stem wood density was calculated for each plot, and used as the response variable in analyses.
Research sample	The sample is 981 plots from mature, structurally-intact and closed canopy forests in tropical and sub-tropical South America. The population is mature, structurally-intact and closed canopy forests in tropical and sub-tropical South America.
Sampling strategy	No procedure was used to predetermine sample size. We use all available plots meeting the criteria described in the data exclusion section. We note that this sample size (981 plots) is substantially larger than previous studies looking at variation in wood density in South American forests, and also includes a wider range of forests as the sample was not restricted to lowland Amazoninan forests.
Data collection	Data were collected by teams of researchers consisting of members of the co-author team and supporting field assistants and botanists. Data collection followed standardized RAINFOR protocols [reference 53 in manuscript]. Where species identification was uncertain samples were collected and compared to herbaria samples. Data were recorded on paper field sheets, and uploaded onto the ForestPlots database.
Timing and spatial scale	We use the first census from each plot as this usually had the highest identification rate. Plots were established between 1957 and 2019, with a median establishment date of 2006. Plot size varied from 0.04 to 25 ha with a mean area of 0.76 ha.
Data exclusions	We excluded (1) data from outside South America, (2) secondary forests, (3) savanna formations, (4) forests with a known history of logging or burning and (5) plots were fewer than 80% of stems were identified to genus level.
Reproducibility	The dataset used comes from extensive and long-running field sampling, and its unique nature means it is not possible to test conclusions on an independent dataset. We did assess model performance using two forms of cross-validation - one randomly selecting plots for inclusion in a training or testing sets, and the other dividing the data into independent groups. Models were also fitted separately to each region, and predictions compared to the global model.
Randomization	Our plot dataset does not result from random sampling, and the logistical difficulties of working in tropical forests means random sampling at this scale is unfeasible. Previous analyses (e.g. ForestPlots 2021, Biological Conservation) indicate that environmental conditions captured by the plot network are representative of the wider area.
Blinding	Blinding is not relevant to this study

Did the study involve field work? ☒ Yes ☐ No

Field work, collection and transport

Field conditions	The environmental gradients sampled by the plot network are provided in Figure S1.
Location	Locations of the 981 plots are shown in Figure 1, and are provided to two decimal places at https://doi.org/10.5521/forestplots.net/2024_4.
Access & import/export	This paper contains data resulting from hundreds of fieldwork campaigns, each led by at least one of the researchers co-authoring this paper, and are the result of a huge community effort and support from numerous agencies.
Disturbance	Our measurements do not cause significant disturbance. We tagged stems with a single nail (aluminum to avoid damage to trees due to corrosion).

Reporting for specific materials, systems and methods

We require information from authors about some types of materials, experimental systems and methods used in many studies. Here, indicate whether each material, system or method listed is relevant to your study. If you are not sure if a list item applies to your research, read the appropriate section before selecting a response.

Materials & experimental systems

n/a	Involved in the study
☒	☐ Antibodies
☒	☐ Eukaryotic cell lines
☒	☐ Palaeontology and archaeology
☒	☐ Animals and other organisms
☒	☐ Clinical data
☒	☐ Dual use research of concern
☒	☐ Plants

Methods

n/a	Involved in the study
☒	☐ ChIP-seq
☒	☐ Flow cytometry
☒	☐ MRI-based neuroimaging

Plants

Seed stocks	This study used wild plants growing in forests without known human influence.
Novel plant genotypes	This study did not involve the production of novel plant genotypes.
Authentication	No seed stock or novel plant genotypes were used.
